# Letter to the Editor on: ‘Association between dental caries and self-reported COPD prevalence in U.S. adults aged ≥60 years: a cross-sectional analysis of NHANES 2015–2020’

**DOI:** 10.1080/20002297.2026.2715818

**Published:** 2026-08-12

**Authors:** Qingqing Shan

**Affiliations:** a Department of Respiration, Chengdu Integrated TCM & Western Medicine Hospital, Chengdu, People’s Republic of China

Dear Editor,

We read with great interest the article by Huang et al. [1], which found significant associations between coronal and root caries and self-reported COPD prevalence in U.S. older adults. While the authors are to be commended for addressing an important knowledge gap in the oral-lung axis, we would like to raise several methodological concerns that justify careful consideration when interpreting the findings.

First, the definition of COPD based solely on three self-reported questionnaire items may substantially compromise internal validity. The study classified participants as having COPD if they answered ‘yes’ to any question regarding prior physician diagnosis of chronic bronchitis, emphysema, or COPD. However, prior validation studies using NHANES spirometry data have demonstrated that questionnaire-based case definitions for COPD show sensitivity as low as 18–35% among smokers when compared against the gold standard of post-bronchodilator spirometry [2]. This low sensitivity indicates that a substantial proportion of true COPD cases—particularly those with milder airflow obstruction—may be misclassified as non-COPD. Conversely, the inclusion of participants with chronic bronchitis who do not meet spirometric criteria for COPD likely introduces false positives. Such non-differential misclassification would typically bias effect estimates toward the null. Still, in the presence of differential recall (e.g. individuals with worse oral health may be more sensitive to respiratory symptoms), spurious associations cannot be excluded. Notably, spirometry data are available for multiple NHANES cycles [3], and their incorporation would have substantially strengthened case ascertainment.

Second, the interpretation within subgroup analyses appears to lack biological plausibility and raises concern about chance findings due to multiple testing. The authors reported that the association between root caries and COPD was most prominent within participants with college education or above (OR = 1.98, 95% CI: 1.10–3.55), whereas no significant association was observed in lower education groups. This surprising finding—that a stronger oral-systemic association exists in a population generally expected to have better oral health literacy and access to dental care—is more plausibly explained by Type I error arising from multiple subgroup comparisons without adjustment for multiplicity. Similarly, the observation that the association between coronal caries and COPD was strongest among former smokers but not current smokers defies straightforward biological explanation. The subgroup findings should be considered hypothesis-generating rather than confirmatory.

Third, the clinical significance of the reported effect sizes deserves reconsideration. A 4% increase in COPD odds per one-unit increment in DMFT (OR = 1.04) is of modest magnitude at best, and the authors did not discuss whether this effect exceeds the minimal clinically important difference. In the absence of a clinically meaningful threshold, the emphasis on statistical significance alone may overstate the real-world implications of the findings.

In brief, while the study supplies an important starting point for investigating the relationship between dental caries and COPD in older adults, the dependence on self-reported case definitions, the lack of adjustment for multiple subgroup comparisons, and the modest effect sizes collectively warrant prudent interpretation. We would value the authors' perspective on whether spirometry data could be used in subsequent analyses to validate these associations, and whether a more conservative analytical approach to subgroup analyses might yield more robust conclusions. Prospective studies with objective pulmonary function testing are clearly needed to establish the temporality and potential causality of these associations.

Sincerely,

Qingqing Shan

Chengdu Integrated TCM & Western Medicine Hospital

## Data Availability

Data sharing does not apply to this article as no data were created or analysed in this study.
